# Current Treatment Options for Cystic Fibrosis-Related Liver Disease

**DOI:** 10.3390/ijms21228586

**Published:** 2020-11-14

**Authors:** Katharina Staufer

**Affiliations:** Department of Visceral Surgery and Medicine, Inselspital, University Hospital Bern, 3010 Bern, Switzerland; Katharina.Staufer@insel.ch; Tel.: +41-31-63-2-74-88

**Keywords:** cystic fibrosis-related liver disease, CFTR modulators, ursodeoxycholic acid, liver transplantation

## Abstract

Cystic Fibrosis-related liver disease (CFLD) has become a leading cause of morbidity and mortality in patients with Cystic Fibrosis (CF), and affects children and adults. The understanding of the pathogenesis of CFLD is key in order to develop efficacious treatments. However, it remains complex, and has not been clarified to the last. The search for a drug might be additionally complicated due to the diverse clinical picture and lack of a unified definition of CFLD. Although ursodeoxycholic acid has been used for decades, its efficacy in CFLD is controversial, and the potential of Cystic Fibrosis Transmembrane Conductance Regulator (CFTR) modulators and targeted gene therapy in CFLD needs to be defined in the near future. This review focuses on the current knowledge on treatment strategies for CFLD based on pathomechanistic viewpoints.

## 1. Introduction

Cystic Fibrosis-related liver disease (CFLD) represents one of multiple optional organ manifestations of Cystic Fibrosis (CF), the most frequent fatal autosomal recessive disorder in Caucasians [1,2]. CF is a monogenic disease resulting from mutations within the cystic fibrosis transmembrane conductance regulator (*CFTR*) on chromosome 7 currently comprising >2000 different mutations [3].

*CFTR* encodes for a protein that is found i.a. in epithelial cells of lungs, sweat glands, pancreas, intestine, and liver. It belongs to the family of ATP-binding cassette (ABC) transporters (CFTR is alternately known as ABC subfamily C member 7 (ABCC7)) and functions as a cyclic adenosine monophosphate (cAMP)-dependent chloride channel that may open on the cell surface of epithelial cells. This chloride channel is necessary to maintain an alkaline pH and dilute fluid secretions [4] via a passive Cl^−^/HCO3^−^ anion exchange on exocrine epithelia along their electrochemical gradient [5,6]. Defects of CFTR may lead to dehydration of secretions and hyperviscous mucus, causing a multisystem disease with major affections of the respiratory, gastrointestinal, and hepatobiliary tract.

## 2. Epidemiology and Clinical Presentation of CFLD

Advances in patient care including solid organ transplantation have led to a strong increase of life expectancy in patients with CF with an estimated median survival of >50 years in patients born in the UK in 2000 and thereafter [7]. The recent introduction of CFTR-directed therapies may further prolong survival in the future.

Albeit lung disease remains the main cause of morbidity and mortality, the changing demographics of CF necessitate an increased focus also on other organ manifestations such as CFLD, which has become one of the leading causes of death among patients with CF [8].

However, the definition and diagnostic criteria of CFLD are subject to adjustments. In 1949 Pugsley and Spence first reported a case of liver cirrhosis in a 17-year old patient with CF [9]. Since then, knowledge has been gathered and refined over years. The definition of CFLD as suggested by Debray et al. in 2011 comprises the presence of significant findings in at least two of the four categories, i.e., physical examination (hepatomegaly and/or splenomegaly), liver function tests (elevated aspartate amino transaminase (AST), alanine amino transaminase (ALT), and gamma glutamyl transferase (GGT) at least at three consecutive determinations over 12 months after excluding other causes of liver disease), ultrasonography (signs of parenchymal liver disease, bile duct dilatation, and/or portal hypertension), and/or liver biopsy [10]. Recently, Koh et al. suggested a refined definition, considering an individual to have CFLD if there is radiologic or histologic evidence of cirrhosis or diffuse liver disease, or a positive finding is present in at least two of four categories, i.e., liver function tests, radiologic imaging, vibration controlled transient elastography, and/or noninvasive fibrosis biomarkers [11]. According to the latter definition of CFLD, also a higher amount of patients affected might be identified [11].

In summary, the clinical presentation of CFLD may include elevated liver enzymes, cholangiopathy, hepatic steatosis, portal hypertension including esophageal varices in about 70%, as well as focal fibrosis and focal cirrhosis [12]. Risk factors for CFLD comprise male sex, presence of severe mutations, history of meconium ileus, exocrine pancreatic insufficiency, Pseudomonas aeruginosa, CF-related diabetes, and carrying the SERPINA 1 Z Allele [13,14,15,16].

The prevalence of CFLD in children and adults ranges from 27 to 47%: In a longitudinal study by Colombo et al. including 177 consecutive patients with CF (mainly children) at their first presentation with a median follow up of 14 years, 27% of patients developed CFLD (incidence rate 1.8%) diagnosed by either ultrasound or liver enzymes (steatosis not deemed as CFLD) [13]. Of these, 35% developed liver cirrhosis (*n* = 17/177 = 9.6%, incidence rate 4.5% during a median period of 5 years after diagnosis of CFLD). In another longitudinal study by Lamireau et al. including 241 children with CF, the prevalence of CFLD (detected by ultrasound) was 41% after 12 years, and cirrhosis occurred in 7.9% (*n* = 19/241) of patients at a median age of 10 years [14].

A study by Koh et al. investigated adult onset CFLD in a cohort of 36 patients without CFLD during childhood, who were followed for up to 38 years (median follow-up 24.5 years) [11]. The prevalence of CFLD was 47% at a median age of 36.6 years.

A further longitudinal population-based study by Toledano et al. analyzed data of 3417 children and adults with CF from the UK CF-registry and showed that the prevalence of CFLD (defined by elevated liver enzymes and/or abnormal liver ultrasound including steatosis) has increased during 2008 to 2013 [16]. Furthermore, mortality in patients with CFLD was more than doubled compared to patients without. Of note, liver cirrhosis was diagnosed at a median age of 19 years.

Thus, one the one hand, childhood-onset CFLD may progress over time, on the other hand also adulthood-onset CFLD may occur with higher age. Both observations may be a result of increased life expectancy of patients with CF. Whether childhood-onset (genetic factors more important) and adulthood-onset CFLD (environmental factors, secondary/tertiary causes more important) have the same pathogenesis in common remains to be elucidated.

Eventually, the definition of CFLD is not unified and is subject to changes due to the changing epidemiology as well as new discoveries that make diagnostic tools more sensitive and specific.

## 3. Pathogenesis of CFLD

The pathogenesis of CFLD is believed to be multifactorial and not clarified to the last [17]. Thinking of the whole clinical picture of CFLD that could be present, i.e., cholangiopathy, steatosis, liver fibrosis, focal cirrhosis, and portal hypertension (without cirrhosis being a mandatory requirement), we probably need to distinguish between primary, secondary, and tertiary causes that also might influence each other.

Primary causes for liver alterations, such as genetic factors affecting bile acid homeostasis and epithelial innate immunity of the bile ducts, secondary causes such as CF-related diabetes (CFRD), exocrine pancreatic insufficiency, intestinal inflammation, changes in the gut microbiome, as well as tertiary causes such as antibiotics, immunosuppressive medication, and high fat diet might coin the clinical presentation of CFLD.

CFTR mutations are classified as class I, II, III, IV, V, and VI depending on the underlying CFTR defect. Class I mutations affect biosynthesis of CFTR and thereby lead to the absence of a functional CFTR protein. Class II mutations (including the most common CFTR mutation deletion of a phenylalanine at position 508 (F508del)) interfere with protein maturation, why CFTR comes up misfolded and causes a CFTR trafficking defect. Class III mutations lead to a defective Cl-/HCO3^−^-channel regulation. Class IV mutations cause a decreased Cl^−^/HCO3^−^-channel conductance, Class V mutations cause a reduced synthesis of CFTR, and Class VI mutations lead to CFTR membrane stability [2].

CFTR genotype-phenotype correlations, however, are generally weak due to different mutation penetrance in various organs. More severe mutations such as classes I, II, and III present with higher morbidity and mortality and correlate well with pancreatic insufficiency (>95%), meconium ileus (>20%), higher sweat chloride levels (≥60 mmol/L), younger age (<1 year) at diagnosis, and liver disease (in 3–5%) [18].

In the liver, CFTR is expressed in the apical membrane of cholangiocytes, but not hepatocytes [19], and constitutes a major determinant of bile secretion. Dysfunctional or lacking CFTR protein leads to a disrupted Cl^−^– and HCO3^−^ secretion, and thus a hyperviscous and more acidic bile (including aberrant bile acid composition and increased concentration of toxic bile acids). The resulting ductular biliary obstruction leads to local inflammation resulting in the picture of sclerosing cholangitis as well as portal inflammation, which may cause a focal fibrosis in the first place, but later multilobular fibrosis and cirrhosis. Furthermore, as recently described by Fiorotto et al., CFTR is additionally involved in the control of biliary epithelial inflammation and permeability in a mouse model [20]. This is facilitated by controlling the activation of the tyrosine kinase Src, a protooncogene, which in turn regulates toll-like receptor 4 (TLR-4)—responses to gut-derived endotoxins. Additionally, aberrant TLR-4 activation decreases the epithelial barrier function leading to a back-diffusion of toxic bile acids again causing peribiliary inflammation and fibrosis [20]. Additionally, the same researchers recently postulated an auto-inflammatory component in the pathogenesis of CFLD as a result of aberrant activation of the innate immune system. This may be mediated via an increased basal NF-kB activation found in human F508del cholangiocytes, which in turn actively secrete pro-inflammatory chemokines known to attract neutrophils. Furthermore, F508del cholangiocytes showed increased responsiveness to endotoxins, as well as Src kinase activation and toll-like receptor 4 (TLR4) phosphorylation leading to local inflammation [21].

Additionally, not only CFTR defects may cause CFLD, but also the SERPINA-1Z–allele has been identified as a modifier gene for the development of CFLD [15]. In patients carrying the Z-allele the α1-antitrypsin variant accumulates in hepatocytes and cholangiocytes [22], which could lead to further endoplasmatic reticulum stress and predispose cholangiocytes to injury of other causes [23]. A recent in silico analysis identified further candidate modifier genes potentially involved in CFLD (i.e., Solute carrier family 33 acetyl-coenzyme A transporter member 1 (SLC33A1), glycoprotein NMB (GPNMB), neutrophil cytosolic Factor 2 (NCF2), RAS guanyl nucleotide-releasing protein 1 (RASGRP1), lectin galactosidase-binding soluble 3 (LGALS3), and protein-tyrosine phosphatase nonreceptor-type 13 (PTPN13)) [24].

A clear definition of CFLD, as well as unraveling the pathomechanism that underlies the evolution of CFLD, including the consideration of its clinical characteristics and severity determine the development of efficacious treatments.

## 4. General Treatment Recommendations for CFLD

Treatment of CFLD should be in the hand of an experienced, multidisciplinary team [25]. It comprises two major aspects, firstly, treating the underlying liver disease itself, and secondly, managing nutritional therapy, as well as portal hypertension and decompensated cirrhosis, both possible results of the underlying liver disease. Liver transplantation may be the ultimate treatment in patients with end-stage liver disease.

Treating the underlying liver disease to date mainly includes the administration of ursodeoxycholic acid (UDCA). Current knowledge on UDCA in CFLD is described in Section 5. The potential of CFTR modulators, gene therapy and future treatment options is described in Section 6, Section 7 and Section 8.

Nutritional therapy with regard to CFLD mainly aims at improving the nutritional status of the patient by optimizing dietary intake of calories (especially protein), pancreatic enzyme replacement, supplementation of fat-soluble vitamins A, D, E, K, and insulin treatment in patients with CFRD. It may also include the correction of deficiencies of essential fatty acids, carnitine and choline since an association with liver steatosis has been postulated [25,26].

The management of portal hypertension in CFLD (especially adulthood CFLD) includes endoscopic screening for gastroesophageal varices and consecutive band ligation or sclerotherapy as primary or secondary bleeding prophylaxis [27]. Unselective beta-blocker therapy with propranolol or carvedilol might be used in order to prevent bleeding [27], but should be used with caution in patients with reactive airway disease, since randomized controlled trials in patients with CF are currently lacking. Other complications of portal hypertension such as ascites, spontaneous bacterial peritonitis and hepatic encephalopathy should be treated according to current guidelines on patients with decompensated liver cirrhosis [28]. However, it needs to be taken into account that portal hypertension in patients with CFLD often occurs in the absence of liver cirrhosis and the presence of preserved liver function [29]. Non-cirrhotic portal hypertension in CFLD was found to be associated with portal branch venopathy in small case series [30,31]. In these patients, transjugular intrahepatic portosystemic shut can be a therapeutic option.

Unified criteria for the indication and timing of liver transplantation for CFLD have not been established so far. However, in patients with CF, deterioration of nutritional status and pulmonary function should be given special consideration [10]. Since the median survival time has been increasing, liver transplantation for CFLD might become more frequent in the future.

## 5. Effects of Ursodeoxycholic Acid on CFLD

As according to the Best Practice Guidance for the Diagnosis and Management of CFLD, UDCA should be commenced as soon as the diagnosis of CFLD has been made at a dose of 20 mg/kg body weight (BW) in order “to delay the progression of the disease” [10].

However, although UDCA has been repeatedly investigated as treatment for CFLD since the 1990s [32,33], until now its efficacy in CFLD has remained controversial. Several small prospective case series and larger retrospective registry data mainly could demonstrate a significant positive effect on liver enzymes, however, randomized controlled trials assessing hard endpoints such as improvement in liver histology, mortality or liver transplant free survival are still lacking but remain crucial. Furthermore, the interpretation of the findings is frequently limited due to the lack of a homogenous definition of CFLD and uniform assessment of liver disease severity. Table 1 summarizes the current knowledge on UDCA in CFLD-treatment.

The lacking evidence is made obvious by a Chochrane review from 2017, which analyzed the present evidence on UDCA in terms of improvement of liver function, risk reduction for the development of chronic liver disease, and improvement in general outcomes in CF [34]. All randomized controlled trials that applied UDCA for at least three months compared with placebo or no additional treatment were considered. Of 12 identified trials only three trials comprising 118 participants dating back to the 1990s were included [35,36], applying 10–20 mg/kg body weight of UDCA for up to 12 months. No significant effect of UDCA could be found, not at least also due to the fact that patient numbers analyzed were very small, and length of treatment with UDCA might have been too short. A more recent longitudinal cohort study by Toledano et al. (already mentioned above) investigating >3000 patients reported a positive association of UDCA with prolonged overall survival in patients without cirrhosis (HR 0.50, 95% CI 0.36–0.69, *p* < 0.0001) in contrast to patients with cirrhotic disease (HR 1.19, 95% CI 0.46–3.10, *p* = 0.71) [16], suggesting a positive effect in patients with mild CFLD.

In summary, although the use of UDCA in CFLD has become a standard treatment, the scientific basis for this requires more reliable data especially for efficacy endpoints in middle-and long-term use. Nevertheless, UDCA appears to be beneficial, especially when started early and against the background of lacking therapeutic alternatives.

## 6. Effects of CFTR Modulators on CFLD

CFTR modulators are small molecules, which directly target CFTR and thereby partly restore altered CFTR protein function. Their development has presented a breakthrough in the treatment of CF, however, their effect on CFLD remains widely unclear.

Generally, we need to distinguish between CFTR potentiators and correctors, as well as stabilizers, amplifiers and read-through agents, which may be given in supplement to the first two. Potentiators keep the CFTR channel open so that chloride transport is ensured, correctors may help to ensure correct CFTR protein folding so that CFTR is able to traffic to the cell surface and remains there longer. In addition, stabilizers promote CFTR maturation and plasma membrane stability, and thereby increase the half-life of the CFTR protein, amplifiers may increase the amount of CFTR protein that is produced by the cell, and read-through agents may bypass premature termination codons. Currently, only CFTR potentiators and correctors are readily available to patients.

The CFTR modulator which was developed first was ivacaftor (VX-770, Kalydeco^®^), a CFTR potentiator. The small molecule treating the class III mutation G551D gained FDA approval in 2012. Later it became apparent that it also works in other class III and IV mutations (gating, residual function, splice and conduction mutations). The second medication which was approved by the FDA in 2015 was a CFTR modulator combination of ivacaftor with lumacaftor (VX-770/VX-809, Orkambi^®^) to treat homozygous F508del mutation (protein processing mutation). However, its effect on improvement of FEV1 and reduction of pulmonary exacerbations was only small to modest, probably also due to antagonistic effects of VX-809 and VX-770. In February 2018 tezacaftor, another corrector, was approved in combination with ivacaftor (VX-661/VX-770, Symdeko^®^) for patients with at least one F508del mutation (protein processing, residual function, splice mutations), bearing the advantage of lesser side effects and drug interactions than the combination of lumafactor/ivacaftor.

Ivacaftor, lumacaftor, and tezacaftor all are deemed as first generation CFTR modulators. Although their development represented a breakthrough in the management of patients with CF, treatment with the first generation CFTR modulators either cannot target frequent mutations, or their efficacy is limited. This has highlighted the need for further drug evolution and led to the development of the next generation CFTR corrector VX-445/ elexacaftor, which is able to correct an additional defect in the formation of the F508del-CFTR protein. Hence, a drug was approved as a triple combination of ivacaftor, tezacaftor, and elexacaftor (VX-770/Vx-661/VX-445, Trikafta™) by the FDA in October 2019. Trikafta™ is approved for patients with at least one F508del mutation and, in contrast to its predecessors, shows higher efficacy and is estimated to be able to treat about 90% of the CF population [50,51,52]. Table 2 presents an overview on approved CFTR modulators and their effects.

Further CFTR modulators currently are being tested in phase 1 or 2, such as the CFTR potentiators VX-561 (deuterated ivacaftor) and ABBV-3067, the CFTR correctors ABBV–2222, and VX-121, the CFTR amplifiers fPTI-428, and PTI-CH, and the read-through agent ELX-02 [53].

For all currently available CFTR modulators in clinics it is recommended to check for liver function test abnormalities (transaminases and bilirubin) on a regular basis, which have been described in about 5% to 15% of patients as a potential side effect of these drugs up to >8 time the upper limit of normal [50,54,55]. However, there is also some evidence that CFTR modulators might have a beneficial effect on the liver. Van de Peppel et al. described that treatment with Ivacaftor partially restored disrupted FGF19-regulated bile acid homeostasis in 117 patients with CFTR gating mutations (partially F508del heterozygoty) participating in the GOAL study, yet these findings did not correlate with CFTR function in other organs, as measured by sweat chloride levels or pulmonary function [56]. Furthermore, mid- and long-term effects on liver function or histology remain unclear. Another study by Gelzo and colleagues reported a beneficial effect of Lumacaftor/Ivacaftor on cholesterol metabolism, enterohepatic flux and improvement of alkaline phosphatase in 40 patients with at least one F508del mutation [57]. A small study including 20 patients with CF, of which nine received Lumacaftor/Ivacaftor, the use of this CFTR modulator combination was associated with less hepatic steatosis as assessed by magnetic resonance imaging proton density fat fraction [58]. In an in vitro study with human derived pluripotent stem cells induced to cholangiocytes, Fiorotto et al. could show that the effect of Lumacaftor/Ivacaftor to correct and potentiate F508del CFTR by a combination with the Src inhibitor PP2 successfully restored fluid secretion to normal levels [21].

Ivacaftor was also hypothesized to be effective in restoring defective phosphatidylcholine secretion in five mutations in the ATP-binding sites of ABCB4, three of which five have been identified as gating mutations in CF (G551D, S1251N, and G1349D) in cell models supplemented by a three-dimensional structural modeling [59].

Finally, Ivacaftor has also shown therapeutic potential in an in vitro model of progressive familial intrahepatic cholestasis type 2 (PFIC2) caused by ABCB11 missense mutations affecting bile salt export pump (BSEP): Ivacaftor treatment increased the taurocholate transport activity of mutated BSEP by 1.7-fold, reaching 95% of BSEP function [60].

In summary, CFTR modulators show a certain therapeutic potential in CFLD, but there is still a long way to go and we are just at the beginning. We need to learn more about the mechanisms underlying hepatic toxicity as well as improvement of liver alterations by CFTR modulators in order to select patients who may benefit. This evolution might be inhibited by the fact that there is no clear association of CFLD with genetic mutations, and that a unified definition of CFLD based on its pathomechanism and pathophysiology is currently lacking. Thus, to date the therapeutic response to CFTR modulators seems unpredictable.

## 7. Effects of Gene Therapies on CFLD

In contrast to CFTR modulators, which partially restore CFTR protein function, *CFTR* targeting gene therapies establish a new, correct version of the *CFTR* gene in order to produce normally functioning CFTR protein. In contrast to CFTR modulators, gene therapy bears the advantage that patients may be treated independently of the underlying *CFTR* mutation.

A randomized controlled trial (phase 2b) investigated a nebulized pGM169/GL67A gene–liposome complex delivering plasmid DNA encoding the *CFTR* gene to the lungs and demonstrated a significant, but modest effect on FEV1% after 1 year of monthly application [66]. Notably, this non-integrating gene therapy had no negative effect on liver enzymes. However, the improvement of lung function was lower in comparison to those achieved with CFTR modulators, so that the substance did not reach clinical application.

Another approach is to use messenger ribonucleic acid (mRNA)-based treatments, such as MRT-5005, to deliver the correct mRNA to lung epithelial cells in order to produce functional CFTR protein. Currently, a clinical phase1/2 trial (NCT03375047, RESTORE-CF) is underway.

Further possibilities evolve by the use of clustered regulatory interspaced short palindrome repeats (CRISPR)/Cas9 to repair defective *CFTR* in vitro and in vivo [67,68,69], but have not been tested in models of CFLD or humans so far.

## 8. Conclusions and Future Treatment Options

There has been tremendous effort and progress in the treatment of CF within the last years. Due to the favorable epidemiologic evolution we have to face increasing numbers of patients suffering from CFLD. However, to date, an efficient causative therapy is lacking.

Besides the evolution of CFTR modulators restoring CFTR protein function, substances creating an additive effect, such as Src inhibitors targeting the TLR-4-mediated inflammatory processes at the bile duct epithelium could be a future treatment option, but have not been investigated in humans. Furthermore, molecules involved in CFTR ubiquitylation that function as regulators of CFTR stability and degradation have been discussed as promising therapeutic targets [70]. Mesenchymal stromal cells and induced pluripotent stem cells have been investigated in CF lung disease in vitro and in vivo, but have not been investigated in CFLD so far [71]. Finally, antifibrotic substances such as Farnesoid X Receptor (FXR) agonists, which target both the gut and the liver, and are centrally involved in bile acid homeostasis might be interesting candidates in the treatment of CFLD, but have not been investigated in this indication [72].

## Figures and Tables

**Table 1:** Ursodeoxycholic Acid as Treatment for Cystic Fibrosis-Related Liver Disease.

Author	Year	Study Design	Number of Patients with CFLD	Mean/Median Age (y)	% Male	Definition of CFLD/Inclusion Criteria	UDCA Dosage (per kg Body Weight/Day)	Treatment Duration (in Months)	Side Effects	Relevant Findings
Cotting et al. [32]	1990	Prospective cohort study without comparison group	8	18.0	62.5	- chronic cholestasis manifested by raised liver enzyme activities over at least one year, associated with abnormal of liver size, liver surface, and homogeneity of the parenchyma in US. - absence of surgically treatable obstruction as a cause of cholestasis shown by US or hepatobiliary scintigraphy with 99mTc labeled N-(2,6-diethyl-3-iodo-phenylcarbamoylmethyl)-iminodiacetate, or both.	15–20 mg	6	none	decrease in AST (*p* < 0.005) and ALT (*p* < 0.001) BW increase (*p* < 0.0001) increase in muscle mass (*p* < 0.05)
Colombo et al. [33]	1990	Prospective cohort study without comparison group	9	10.7	66.0	- presence of firm hepatomegaly and - persistent increase in standard liver function test values during a 2-year period	10–15 mg (plus taurine 30 mg/kg BW)	6	minor abdominal discomfort in one patient	decrease in ALT and GGT (*p* < 0.01) and AP (*p* < 0.05)
Colombo et al. [37]	1992	Prospective cohort study without comparison group	13 (of these 9 patients were already included in [24])	11.0	69.2	- presence of clinical, biochemical and abnormalities in US	15–20 mg	10 to 12	none	improvement in canalicular excretory function and biliary drainage (hepatobiliary scintigraphy with 99mTc-labeled trimethyl-bromo-iminodiacetic acid)
Galabert et al. [38]	1992	Prospective cohort study without comparison group	22	15.3	77.3	- existence of chronic cholestasis assessed by a persistent (>1 year) and significant increase (>2 upper normal limit) of GGT, ALP and 5′-nucleotidase - presence of firm hepatomegaly, and - abnormal findings on US (increased size, nonhomogeneous echogenicity, irregular surface)	10–20 mg	12	none	decrease in ALT (*p* < 0.002), AST (*p* < 0.01), GGT (*p* < 0.001), AP (*p* < 0.001)
Colombo et al. [39]	1992	Prospective cohort study without comparison group	9	11.8	77.8	- progressive liver disease was documented over a 2-yr period in all patients from clinical, biochemical and echographical abnormalities	5, 10, and 15 mg in a replicated latin square design for 2 mo, followed by 20 mg for 2 mo	4 (2 mo plus 2 mo)	n.r.	biochemical improvement in serum liver enzymes was significantly greater with higher doses
O’Brien et al. [40]	1996	Posthoc analysis of randomized controlled trial	6 vs. 6 without UDCA	n.r.	n.r.	- hepatomegaly (liver span in excess of 12 cm) and splenomegaly or both, confirmed by US and - abnormal liver biochemistry (GGT >50 IU/l, 5′nucleotidase > 15 IU/I) or - both for at least 6 mo	20 mg	6	n.r.	decrease in AST, GGT, and 5′nucleosidase (*p* < 0.05)
Colombo et al. [35]	1996	Multicentre, double-blind, placebo-controlled trial	55	13.8	70.9	- presence of hepatomegaly, confirmed by US (increased liver size, nonhomogeneous echogenic pattern, and irregular surface), and the presence of abnormal liver biochemistries (serum transaminases, GGT) of at least 1 year’s duration. - presence of serum transaminase and GGT levels exceeding 1.5 times the upper limit of normal on at least three determinations over the year	15–20 mg (+ 30 mg/kg BW taurine) UDCA + taurine vs. UDCA + placebo vs. placebo + taurine vs. double placebo	12	transient diarrhea, mild and transient abdominal pain	decrease in GGT (*p* = 0.025)
Lepage et al. [36]	1997	Randomized double-blind placebo-controlled crossover study	19	11.9	68.4	- abnormal findings on at least two liver function tests (ALT, AST, GGT) and - an abnormal finding on US or liver biopsy or both	15 mg; increased to 30 mg if less than 50% decrease of ALT or AST or both within 2 mo was achieved (UDCA vs placebo)	6 (6 mo placebo, 6 mo UDCA; 13 of 19 pts followed for 25 mo)	none	decrease in ALT (*p* < 0.01) and GGT (*p* < 0.001)
Van de Meeberg et al. [41]	1997	Randomized trial (low dose vs. high dose UDCA)	30	18.3	64.7	- disturbed liver biochemistry and the presence of abnormalities in US or esophageal varices on endoscopy at two separate occasions at least 3 mo apart - two or more of the following biochemical variables > 1.5 the upper normal limit: bilirubin, AP, GGT, ALT, AST, or - if one of these factors > 1.5 the upper normal limit in combination with the presence of splenomegaly or esophageal varices	10 mg vs. 20 mg	12	severe pruritus in 2 pts required treatment stopp	- decrease in ALT (*p* < 0.02) and GGT (*p* < 0.004) - high dose UDCA induces a better response in liver enzymes
Lindblad et al. [42]	1998	Prospective cohort study without comparison group	10	17.6	60.0	- probable or proven cirrhosis on liver biopsy, including fibrosis with extensive bridging and/or signs of irregularities of the intrahepatic bile ducts at endoscopic retrograde cholangiography	10–15 mg	24	none	- decrease in ALT and AST (*p* < 0.05) - decrease in GGT only significant after 12 (*p* < 0.05) but not 24 mo - decrease in immunoglobuline G (*p* < 0.01)
Colombo et al. [43,44]	1999	Prospective cohort study without comparison group	36	10.0	69.4	- persistence of at least two of the following clinical, laboratory and ultrasonographic signs for more than 1 year: hepatomegaly, elevated levels of serum liver enzymes (AST, ALT, GGT), abnormalities in US (hepatomegaly, increased or heterogeneous echogenicity, nodularity, irregular margins, or splenomegaly). - exclusion of other liver diseases by evaluating copper metabolism, α1-antitrypsin, non-organ specific autoantibodies and serologic markers of hepatitis B and C infection	20mg	60.5	n.r.	- decrease in AST and GGT (*p* ≤ 0.0125) - delayed intestinal visualization at hepatobiliary scintigraphy predicts better response to UDCA
Nousia-Arvanitakis et al. [35]	2001	Prospective cohort study without comparison group	7	n.r.	n.r.	- pts with nodular biliary cirrhosis: hepatosplenomegaly, abnormal liver function tests, and micronodular, multilobular heterogeneous hyperechogenicity of the hepatic parenchyma in US confirmed by percutaneous liver biopsy	20mg	120	n.r.	- normalization of ALT, AST and GGT in all pts - no effect on focal biliary cirrhosis
Desmond et al. [45]	2007	Retrospective cohort study	22	n.r.	63.0	- persistent (> 6 mo) and significant (> ×2 upper normal limit) AST, ALT and/or ALP elevation and/or hepatobiliary symptoms (pruritus, fatigue or right upper quadrant pain) - positive liver histology (focal biliary cirrhosis or multilobular cirrhosis), or at least two of the following parameters on at least two consecutive examinations spanning a 1-year period: (i) clinical hepatomegaly (liver span 412 cm in the midclavicular line), confirmed by US; (ii) abnormal serum liver enzyme levels, consisting of elevation above the upper normal limits of two of the following: AST, ALT, GGT or AP; and (iii) US abnormalities other than hepatomegaly (i.e., increased, heterogeneous echogenicity; nodularity; irregular margins; splenomegaly). Ultrasonographic pattern of steatosis did not represent a diagnostic criterion. At both the paediatric hospital and the adult hospital, patients were screened for other causes of abnormal liver enzyme levels (i.e., hepatitis A, B or C virus; cytomegalovirus; Epstein–Barr virus; alcohol; drugs; or toxins) and were excluded if any of these were present	10–15 mg was titrated upwards according to symptomatic and/or biochemical response (reduction in liver enzymes to < ×1.5 upper normal limit)	43.2	transient pruritus in 2 pts	- decrease in ALT (*p* < 0.001), AST (*p* = 0.005), GGT (*p* = 0.021) and AP (*p* < 0.001) - improvement in hepatobiliary symptoms including pruritus, fatigue and right upper quadrant pain (*p* = 0.0003)
Siano et al. [46]	2010	Retrospective cohort study	26 (14 pts with early vs. 12 pts with late introduction of UDCA	n.r.	50.0 vs. 75.0	- clinical (hepatomegaly), biochemical (increase of at least two serum liver enzyme levels above the upper normal limit) or abnormalities in US (increased echogenicity, no cirrhosis) recorded on two consecutive examinations within a 3-month period, in the absence of other possible causes - US pattern of steatosis did not represent a selection criterion	15 mg	108	n.r.	lower prevalence of CFLD in early UDCA group (*p* < 0.05)
Kappler et al. [47]	2012	Retrospective case-control study	98 vs. 98 age- and gender-matched controls without CFLD vs. 9 historical controls with CFLD	14.8	57.1	- elevation of one or more serum liver enzymes (ALT, AST, GGT, GLDH) > 1.5 upper normal limit and persisting for > 6 months, or - liver enlargement >2 cm for > 6 mo - other potential causes of liver disease such as alpha 1-antitrypsin deficiency excluded	20 mg	86.4	n.r.	decrease in ALT, AST, GGT, GLDH (*p* < 0.001)
Van der Feen et al. [48]	2016	Retrospective cohort study	32 vs. 73 patients without CFLD	10.3	62.5	- at least two of the following conditions were present: hepatomegaly confirmed by US, other abnormalities of the liver parenchyma on ultrasound, e.g., heterogeneous echogenicity and persistently increased liver enzymes (ASAT, ALAT, GGT) with at least two out of these three being abnormal for at least 12 mo - persistently raised transaminases only - US examination consistent with cirrhosis, i.e., nodular aspect of the liver and/or a clearly irregular liver contour and/or splenomegaly	15–20 mg	103.2	n.r.	- decrease of ALT (*p* < 0.001), AST (*p* < 0.01), and GGT (*p* = 0.01) - Decrease in liver stiffness (*p* < 0.01) in a subgroup of patients
Boelle et al. [49]	2019	Retrospective multicentre cohort study	605 (of these 38% under UDCA at the age of 30) vs. 2723 without CFLD	17.9	56.5	- at least two of the following characteristics present: (1) abnormal physical examination (hepatomegaly and/or splenomegaly); (2) abnormalities of liver function tests defined as an increase of transaminase (ALT and/or AST) and/or GGT levels above the upper normal limits; (3) US evidence of liver involvement (heterogeneous echogenicity, irregular margins, or nodularity), portal hypertension (splenomegaly, increased thickness of the lesser omentum, spontaneous splenorenal anastomosis, large collateral veins, or ascites), or biliary abnormalities (bile duct dilatation) - cirrhosis, diagnosed by US, CT, and/or MRI, and/or portal hypertension (splenomegaly, hypersplenism (platelets <150 G/L and white blood cells <3 G/L), and/or spontaneous portosystemic shunts on US) and/or esophageal varices	n.r.	n.r.	n.r.	no evidence of a change in mean age at severe CFLD onset with UDCA treatment
Toledano et al. [16]	2019	Retrospective multicentre cohort study	1749 on UDCA vs. 1668 without UDCA	21.3	58.6	- elevated liver enzymes (ALT, AST, or GGT) - abnormal (but non-cirrhotic) appearances in US including steatosis - evidence of cirrhosis in US - evidence of cirrhosis and portal hypertension as evidenced by the presence of splenomegaly, ascites or gastro-oesophageal varices	n.r.	48	n.r.	UDCA in non-cirrhotic pts associated with prolonged overall survival (HR0.50, 95% CI 0.36–0.69, *p* < 0.0001), but not in cirrhotic pts (HR 1.19, 95% CI 0.46–3.10, *p* = 0.71)

ALT, alanine aminotransferase; AST, aspartate amino transferase; AP, alkaline phosphatase; BW, body weight; CT, computed tomography; GGT, gamma glutamyl transpeptidase; GLDH, glutamate dehydrogenase; MRI, magnetic resonance imaging; n.r., not reported; pts, patients; UDCA, ursodeoxycholic acid; US, ultrasound; Tc; technecium.

**Table 2:** Effects of approved Cystic Fibrosis Transmembrane Conductance Regulator modulators on Cystic Fibrosis organ manifestations.

CFTR Modulator	Effect on CFTR	Targeted CFTR Mutation	Effect on the Liver	Effect on other Organ Systems	References
ivacaftor (VX-770)	potentiator	G551D, G1244E, G1349D, G178R, G551S, S1251N, S1255P, S549N und S549R	Increase in ALT (13.2%), AST (9.6%) and bilirubin (2.4%) > 2x to > 8x ULN	Significant improvement in FEV1% after 24 and 48 weeks Significant reduction of pulmonary exacerbations Significant increase in body weight	[54] (adults)
			Elevate of liver function test > 8x ULN (15%)	significant improvement in FEV1% after 24 and 48 weeks significant reduction of pulmonary exacerbations, significant increase in body weight	[55] (small children)
			rescue of disease-causing variations in ATP-binding sites of ABCB4 that cause defects in phosphatidylcholine secretion relevant to bile composition		[59]
			restores FGF19 regulated bile acid homeostasis		[56]
				increase in small intestinal pH	[61]
				increase in *Akkermansia*, decrease in intestinal inflammation	[62]
ivacaftor/lumacaftor (VX-770/VX-809)	potentiator/corrector	Homozygous F508del	One patient discontinued treatment because of abnormal liver function tests (before taking lumacaftor)	Significant improvement in FEV1% after 56 days	[63]
			significantly lower hepatic steatosis measured by magnetic resonance imaging proton density fat fraction		[58]
ivacaftor/tezacaftor (VX-770/VX-661)	potentiator/corrector	Hetero- and homozygous F508del	Elevation of transaminases >3x ULN (0.6%) Elevation of bilirubin >2x ULN (1.2%)	significant improvement in FEV1% after 56 days	[64]
		Heterozygous F508del		significant improvement in FEV1% after 8 weeks	[65]
ivacaftor/tezacaftor/elexacaftor (VX-770/Vx-661/VX-445	potentiator/corrector/corrector	Hetero- and homozygous F508del	Increased liver enzymes (8%) and bilirubin (3%) > 2x ULN	Significant increase in FEV1% through day 29	[50]
			increase in aminotransferase concentrations (12%) >3x to >5x ULN	significant increase in FEV1% through week 4, significant increase in body weight	[52]

## Abbreviations

CF	Cystic Fibrosis
CFLD	Cystic Fibrosis-related liver disease
CFTR	cystic fibrosis transmembrane conductance regulator
ABC	ATP-binding cassette
ABCC7	ABC subfamily C member 7
AST	aspartate amino transaminase
ALT	alanine amino transaminase
GGT CFRD	gamma glutamyl transferase Cystic Fibrosis–related diabetes mellitus
TLR4	toll-like receptor 4
SLC33A1	Solute carrier family 33 acetyl-coenzyme A transporter member 1
GPNMB	glycoprotein NMB
NCF2	neutrophil cytosolic Factor 2
RASGRP1	RAS guanyl nucleotide-releasing protein 1
LGALS3	lectin galactosidase-binding soluble 3
PTPN13	protein-tyrosine phosphatase nonreceptor-type 13
UDCA	ursodeoxycholic acid
BW	body weight
FEV1	forced expiratory volume in one second
FXR	Farnesoid X Receptor
